# Clinical Audit of Dementia Diagnosis and Management – According to Disease Severity

**DOI:** 10.1192/bjo.2024.568

**Published:** 2024-08-01

**Authors:** Sylvia Fatunla

**Affiliations:** Tees Esk and Wear Valleys NHS Foundation Trust, Middlesbrough, United Kingdom

## Abstract

**Aims:**

The NICE guidelines NG97 (1.5) currently recommend to consider memantine in addition to acetylcholinesterase inhibitors in the management of moderate to severe Alzheimer's dementia, if tolerated, as opposed to the mono-therapeutic management with either class of drug. This management practice was adopted by the trust and updated on Trust guidelines.

I noted that most of the patients on follow up in memory clinic, had a generic diagnosis of dementia subtypes, without a mention of the degree of severity of the illness and as such, on monotherapy.

Aim

This audit serves to establish the practice in recent years, if patients diagnosed with dementia, were diagnosed, and managed according to the severity of their condition.

If not, for necessary changes to be implemented in practice and a re-audit carried out.

**Methods:**

The audit was conducted in February 2023 as a retrospective study. We analysed records of 60 patients seen for a diagnostic appointment in Middlesbrough Memory Service between January 2020 and December 2020.

All referrals made to the memory clinic within 2020 were retrieved from trust electronic records and 5 patients were selected at random from each month using the google random number generator, and analysed on Excel.

**Results:**

The most common dementia diagnosis was mixed dementia (Alzheimer's + vascular disease) with 40% of diagnoses, followed by Alzheimer's disease at 39%, while Lewy body dementia was least diagnosed at 8%.

**Assessment:** Only 46 records completed the dementia diagnostic pathway (initial assessment, ACE III, CT scan alongside pre-referral blood screen), the other 14 patients were unable to complete this pathway due to functional decline.

**Severity of illness:** Of the patients evaluated, only 7% had the severity of disease in their diagnosis, which were all Moderate severity. 93% had generic diagnosis.

**Pharmacological Treatment:** 46 out of 60 patients evaluated, were on medication.

And all were on monotherapy, irrespective of disease severity, with majority being on anticholinesterase inhibitors (Donepezil) being the first and most popular choice.

**Conclusion:**

Severity of disease condition were not identified or documented.

The use of combination therapy is yet to be considered at the diagnostic stage. This should be implemented before discharging a patient with moderate to severe disease. Although, local best practice is to offer this as early as moderate disease is identified.

Combination therapy is yet to be adopted in the organic pathway.

